# Supplementary material for: Genome-Wide Identification and Expression Pattern Analysis of Dirigent Members in the Genus Oryza
Source: Int J Mol Sci. 2023 Apr 13;24(8):7189. doi: 10.3390/ijms24087189 (PMC10138954; doi:10.3390/ijms24087189)
Supplement: Supplementary file 1 [file ijms-24-07189-s001.zip › Supplemental Figures S1-S4.- Dirigent.pdf]

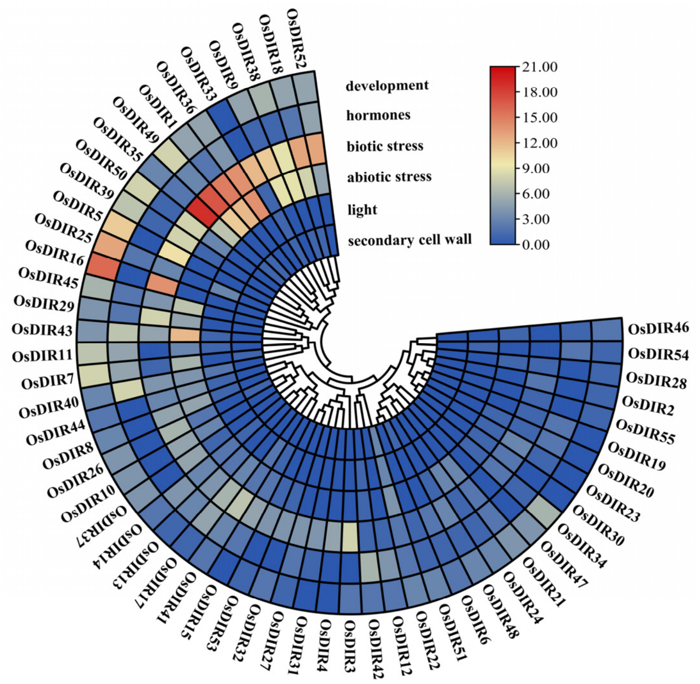

Supplemental Figure S1. Identification of transcription factor binding sites (TFBS) in the promoter regions of *OsjDIR* genes. A total 86 JASPAR matrices were selected. P-value ≤ 1E^{-7}. Heat map represent the number of TFBS in each variety. Including secondary cell wall, light, abiotic stress, biotic stress, hormones and development. The heatmap was constructed by Tbtools software.

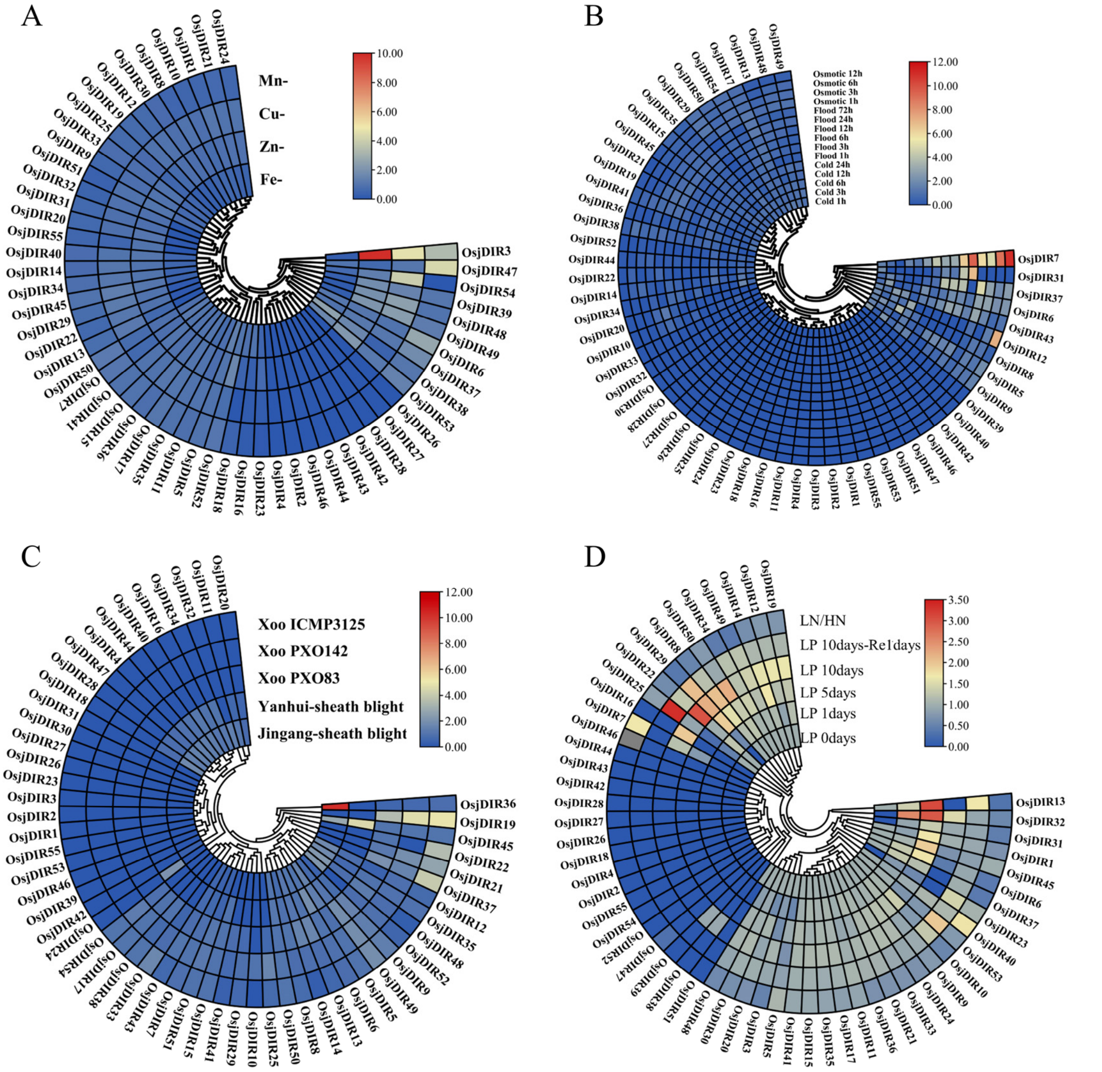

Supplemental Figure S2. Expression profiles of the *OsjDIR* genes in response to the biotic and abiotic stresses based on the RNA-seq data. (A) The heat map exhibited the relative expression levels of *OsjDIR* genes in response to nutrient elements deficiency (Mn-, Cu-, Zn-, Fe-) treatments. (B) The heat map exhibited the relative expression of *OsjDIR* genes in response to osmotic, flood and cold stress. (C) The heat map exhibited the expression of *OsjDIR* genes in response to Xoo and sheath blight infection. (D) The heat map exhibited the expression levels of *OsjDIR* genes under low nitrogen (LN) and low phosphate (LP) treatments. The heatmap was constructed by Tbtools software.

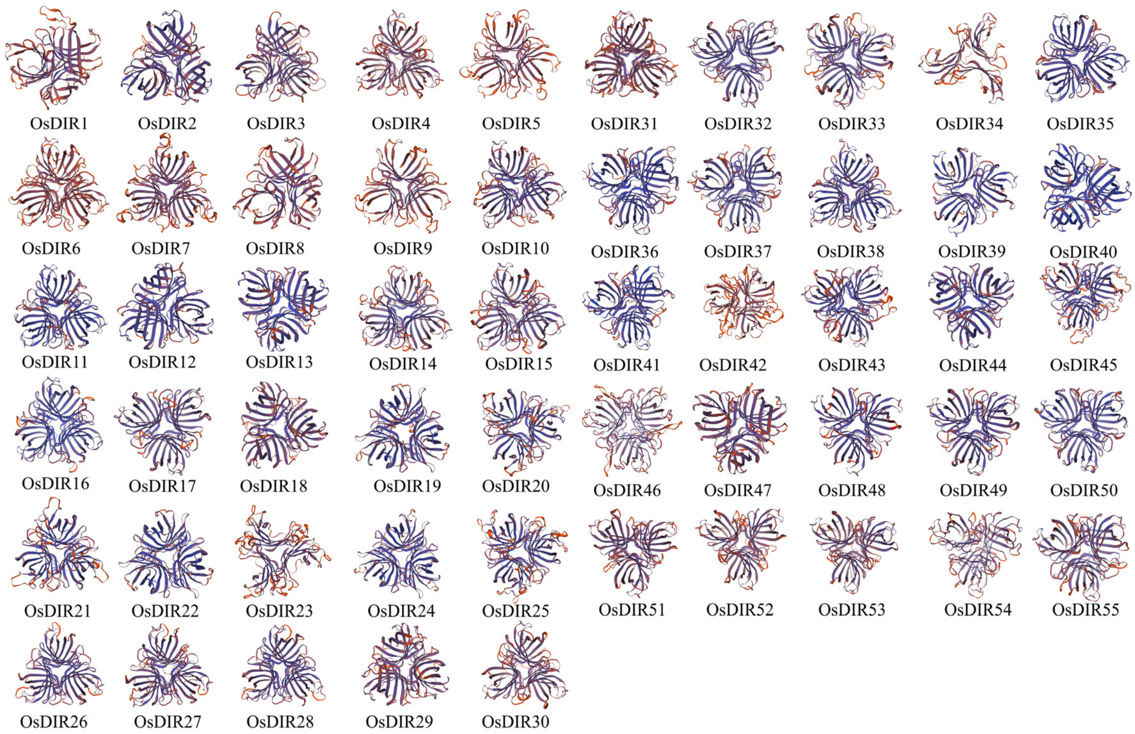

Supplemental Figure S3. Predicted tertiary structures of rice OsjDIR proteins. The tertiary structures of DIR protein from *Oryza sativa* ssp. *Japonica* were predicted by the SWISS website (https://swissmodel.expasy.org/interactive, accessed on 5 April 2022).

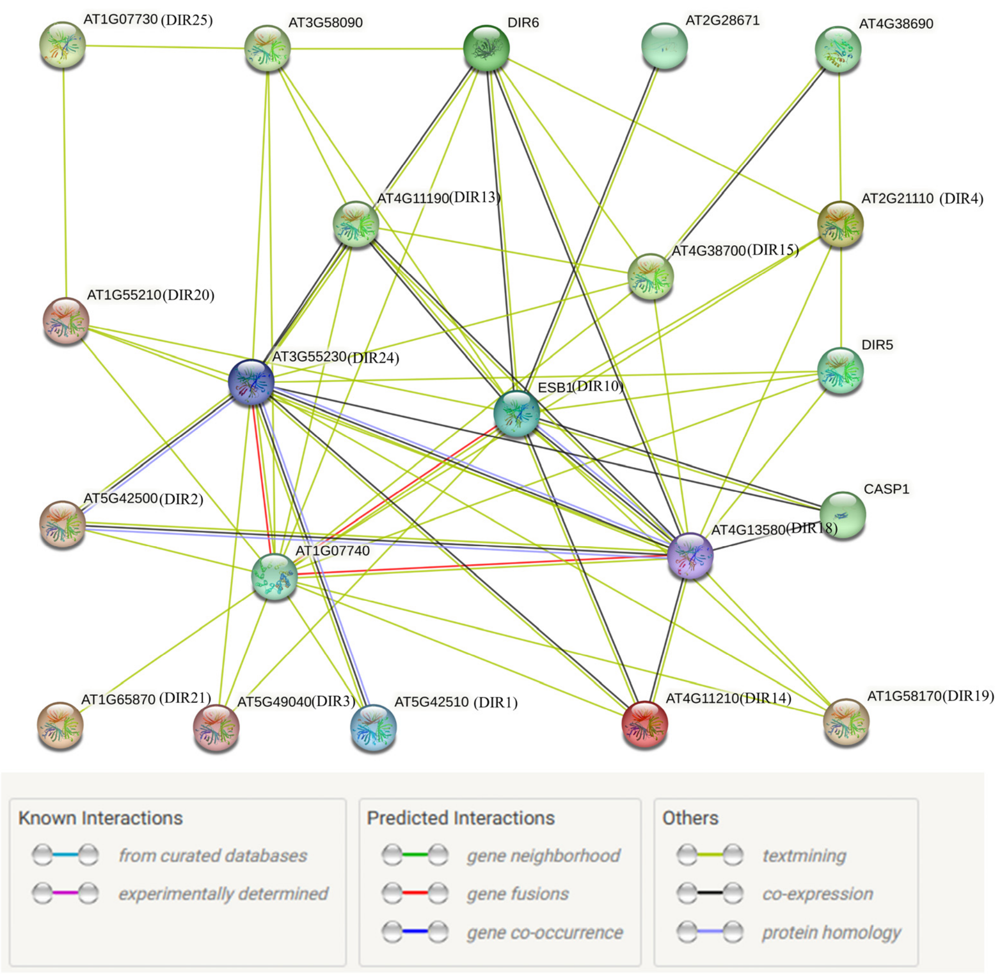

Supplemental Figure S4. Predicted interaction networks of dirigent proteins in Arabidopsis. A protein–protein interaction network among dirigent proteins in Arabidopsis was predicted using STRING (https://cn.string-db.org/, accessed on 20 April 2022).
