# Supplementary figures and images for: Genome-Wide Identification and Expression Pattern Analysis of Dirigent Members in the Genus Oryza
Source: Int J Mol Sci. 2023 Apr 13;24(8):7189. doi: 10.3390/ijms24087189 (PMC10138954; doi:10.3390/ijms24087189)

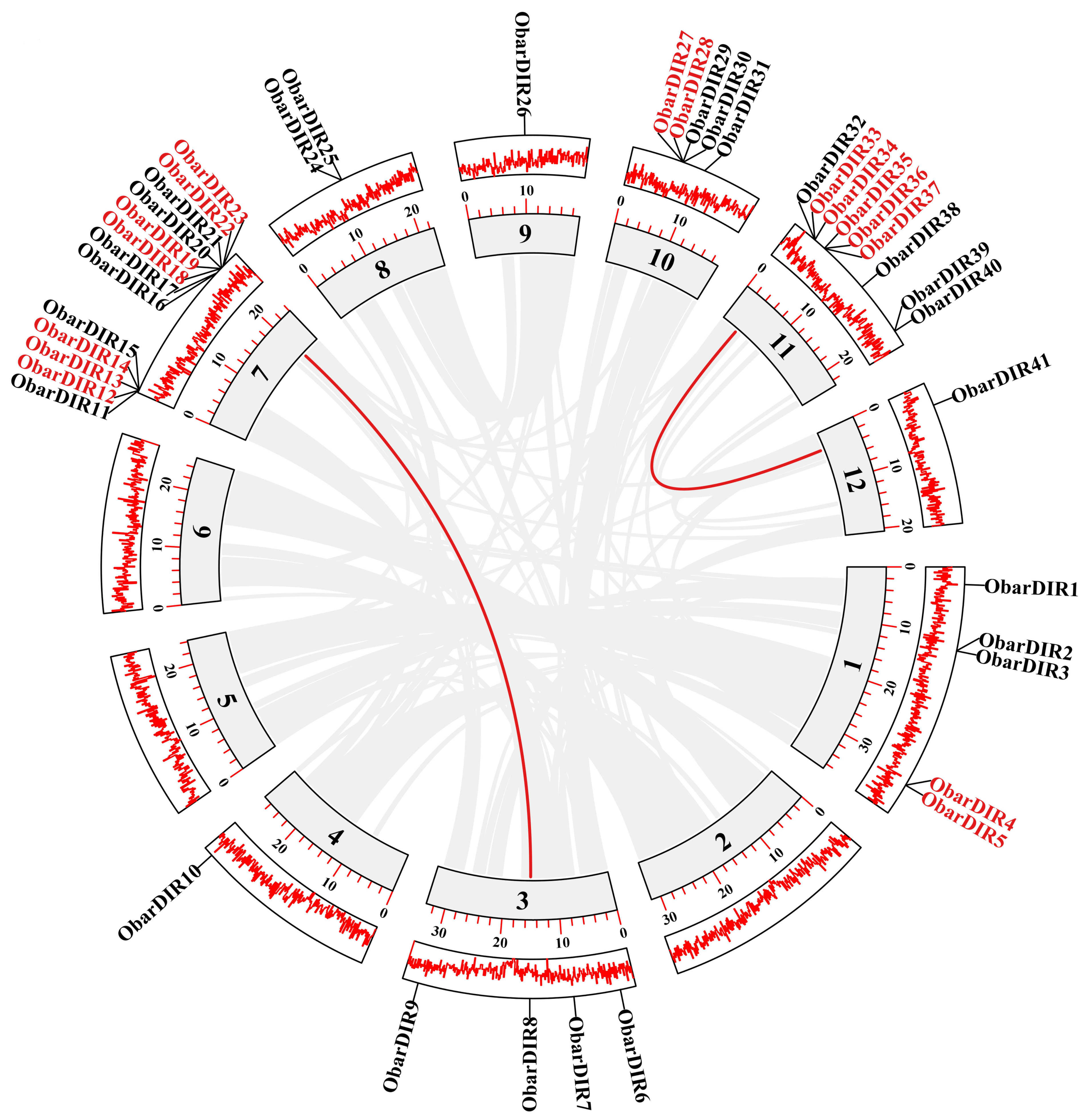

Supplement: Supplementary file 1 [file ijms-24-07189-s001.zip › The location and duplication events of the DIR genes in the genus Oryza/obar.tif]

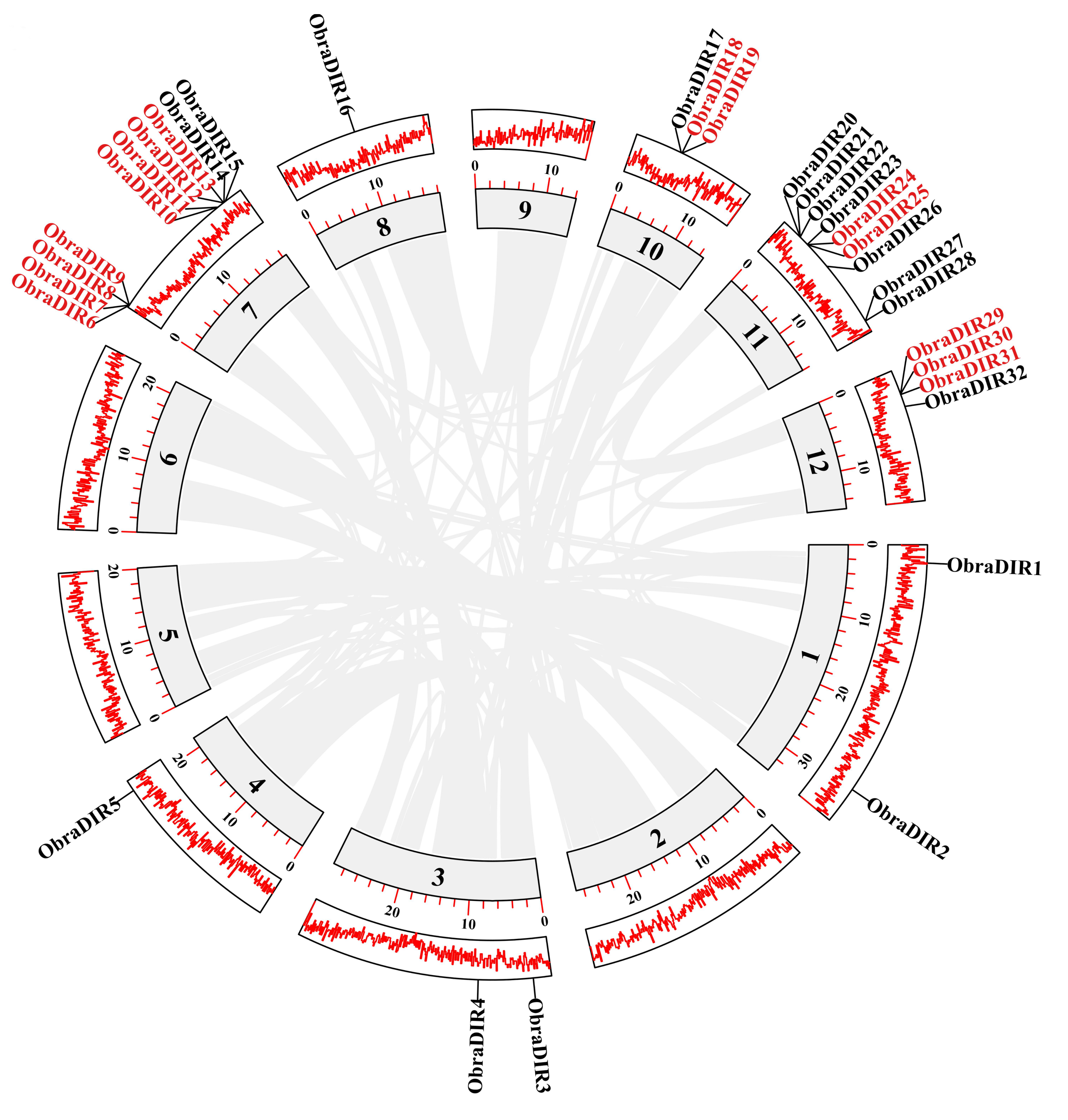

Supplement: Supplementary file 1 [file ijms-24-07189-s001.zip › The location and duplication events of the DIR genes in the genus Oryza/obra.tif]

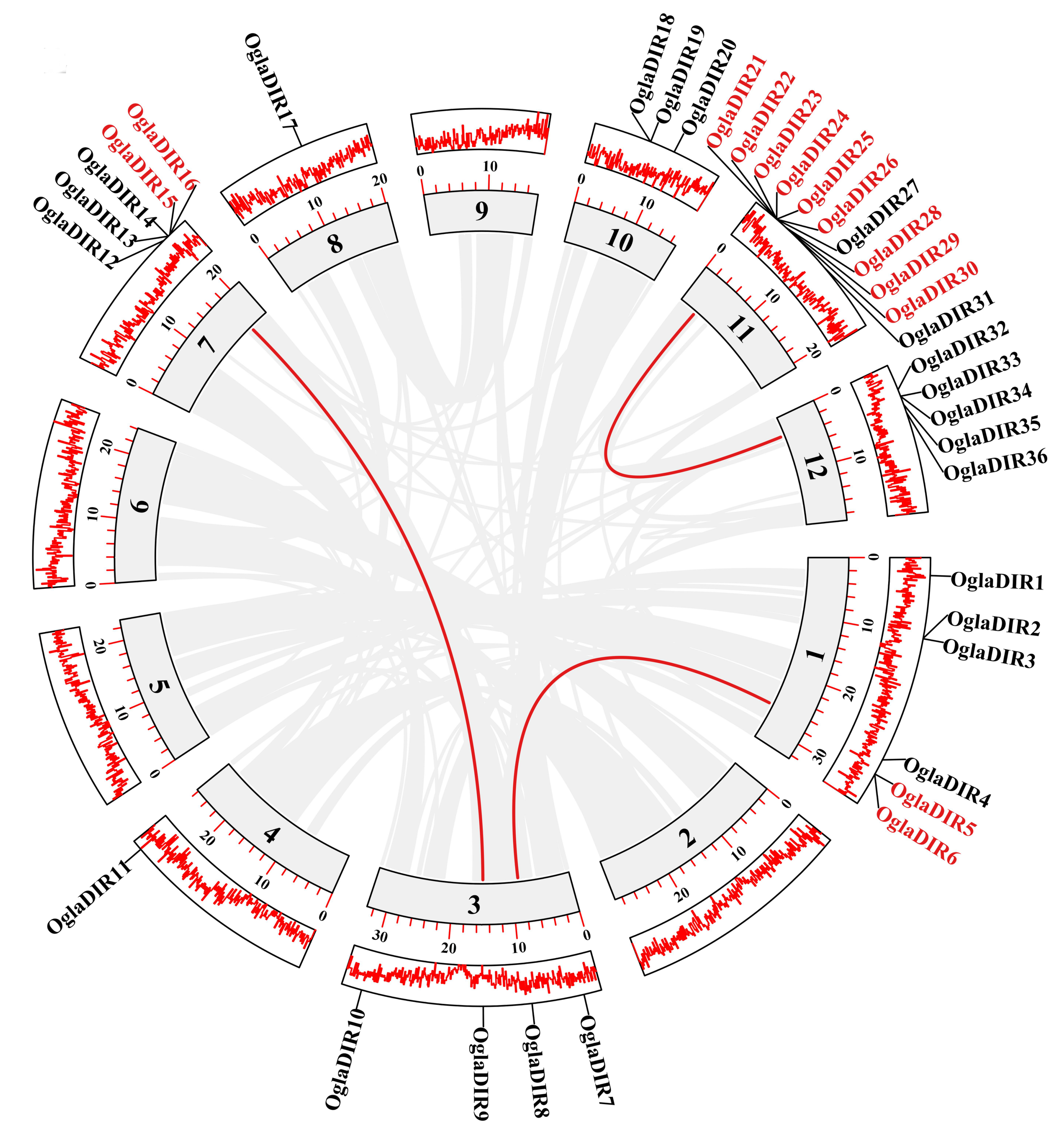

Supplement: Supplementary file 1 [file ijms-24-07189-s001.zip › The location and duplication events of the DIR genes in the genus Oryza/ogla.tif]

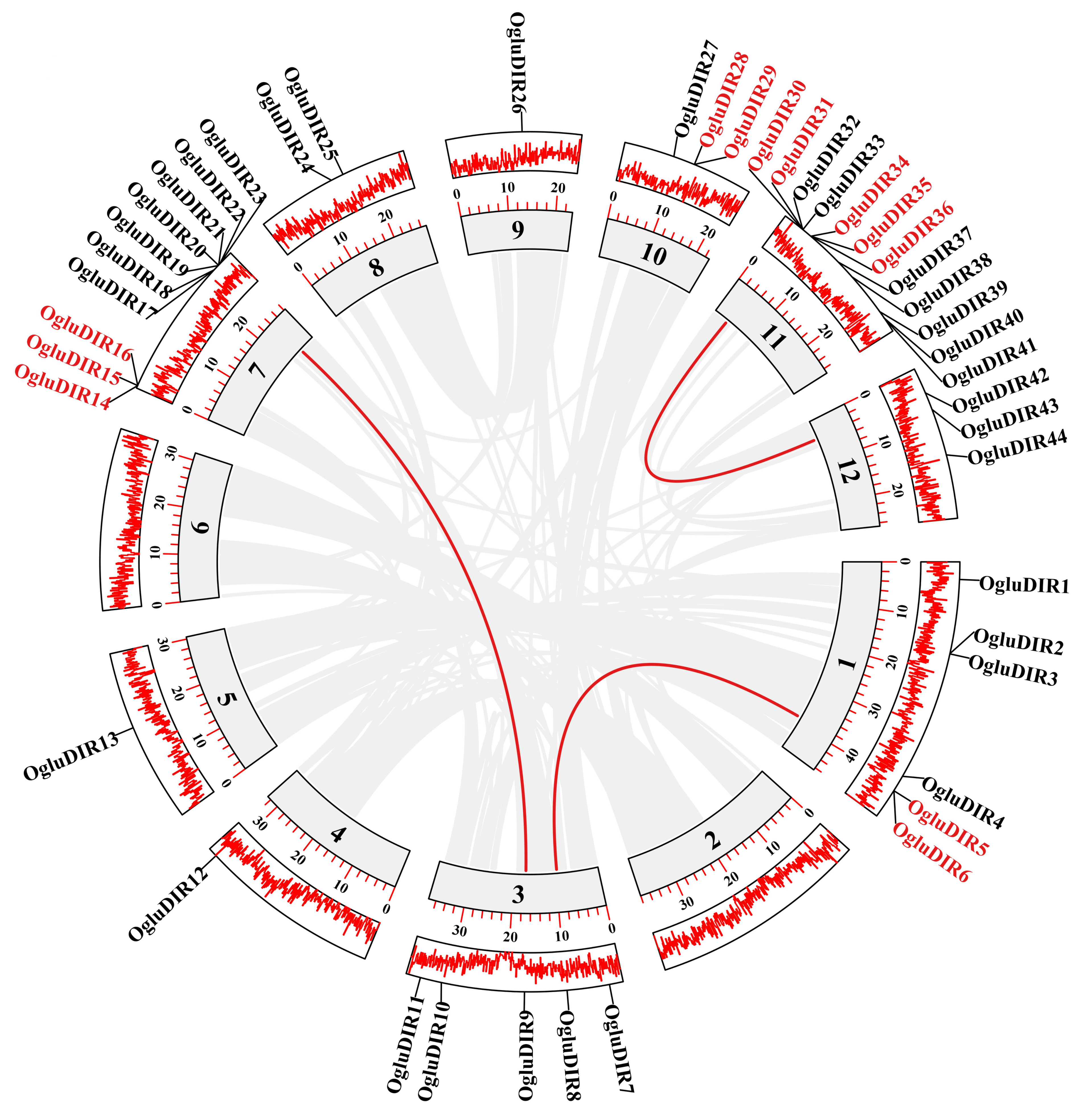

Supplement: Supplementary file 1 [file ijms-24-07189-s001.zip › The location and duplication events of the DIR genes in the genus Oryza/oglu.tif]

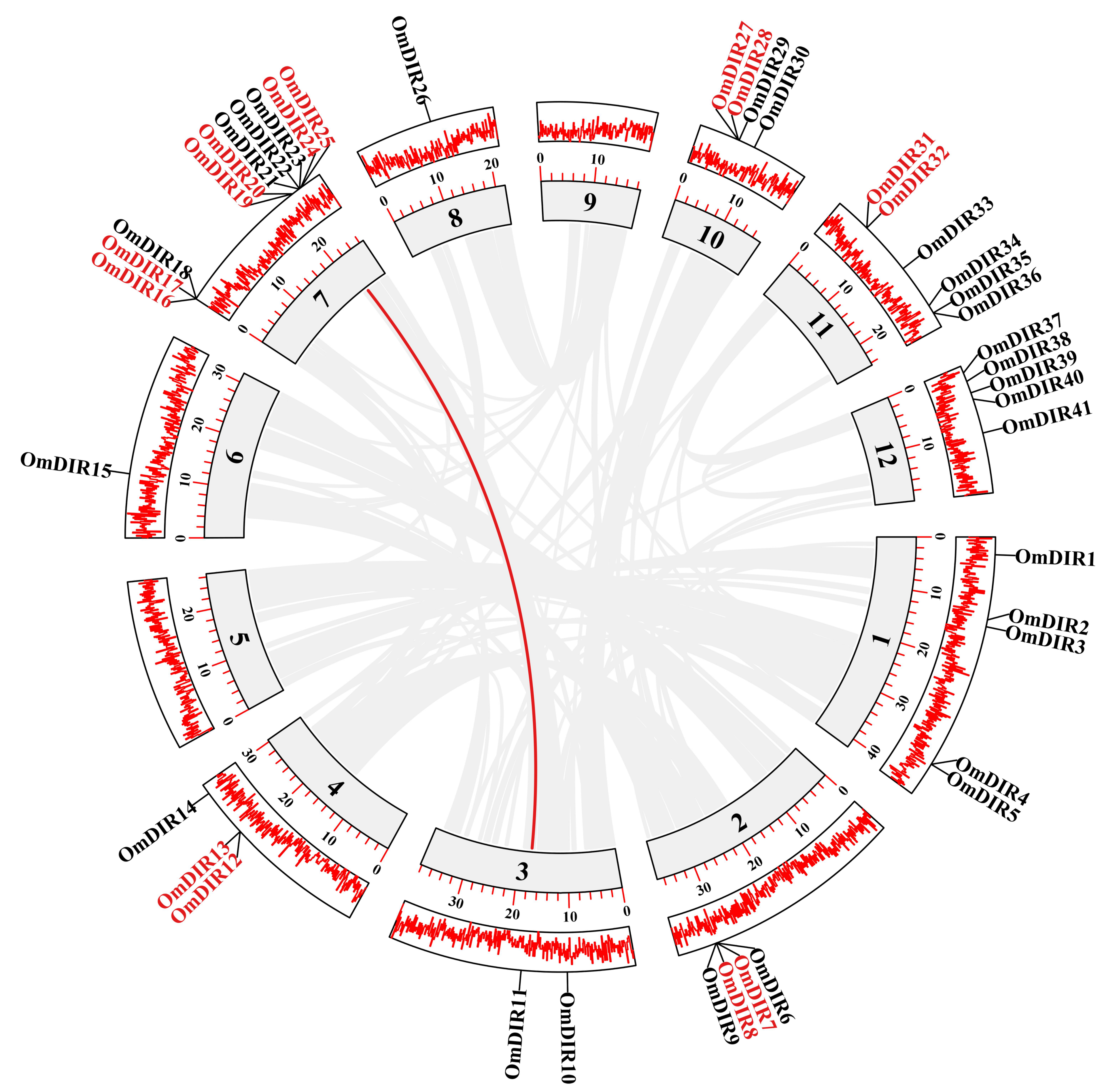

Supplement: Supplementary file 1 [file ijms-24-07189-s001.zip › The location and duplication events of the DIR genes in the genus Oryza/omer.tif]

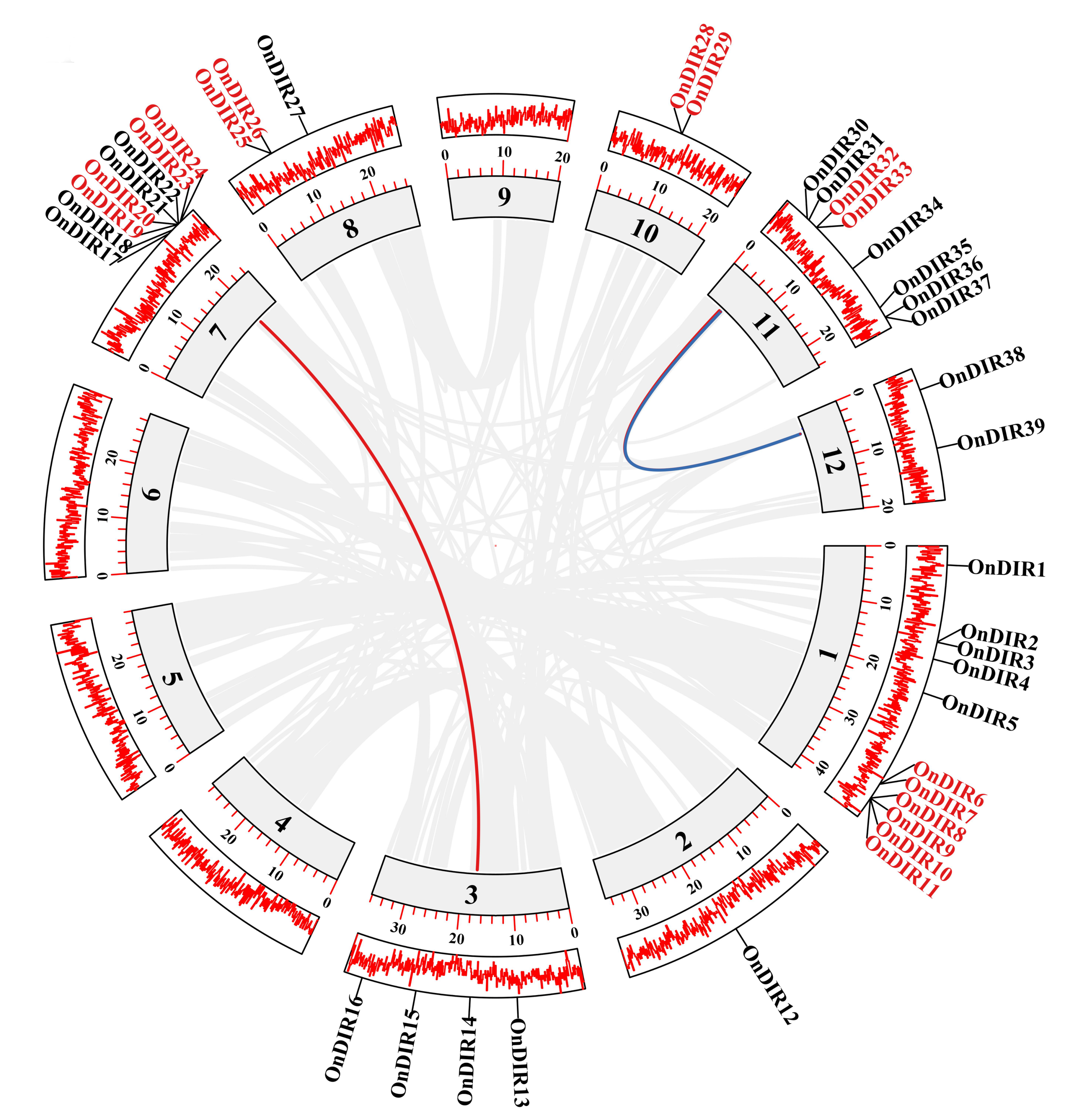

Supplement: Supplementary file 1 [file ijms-24-07189-s001.zip › The location and duplication events of the DIR genes in the genus Oryza/on.tif]

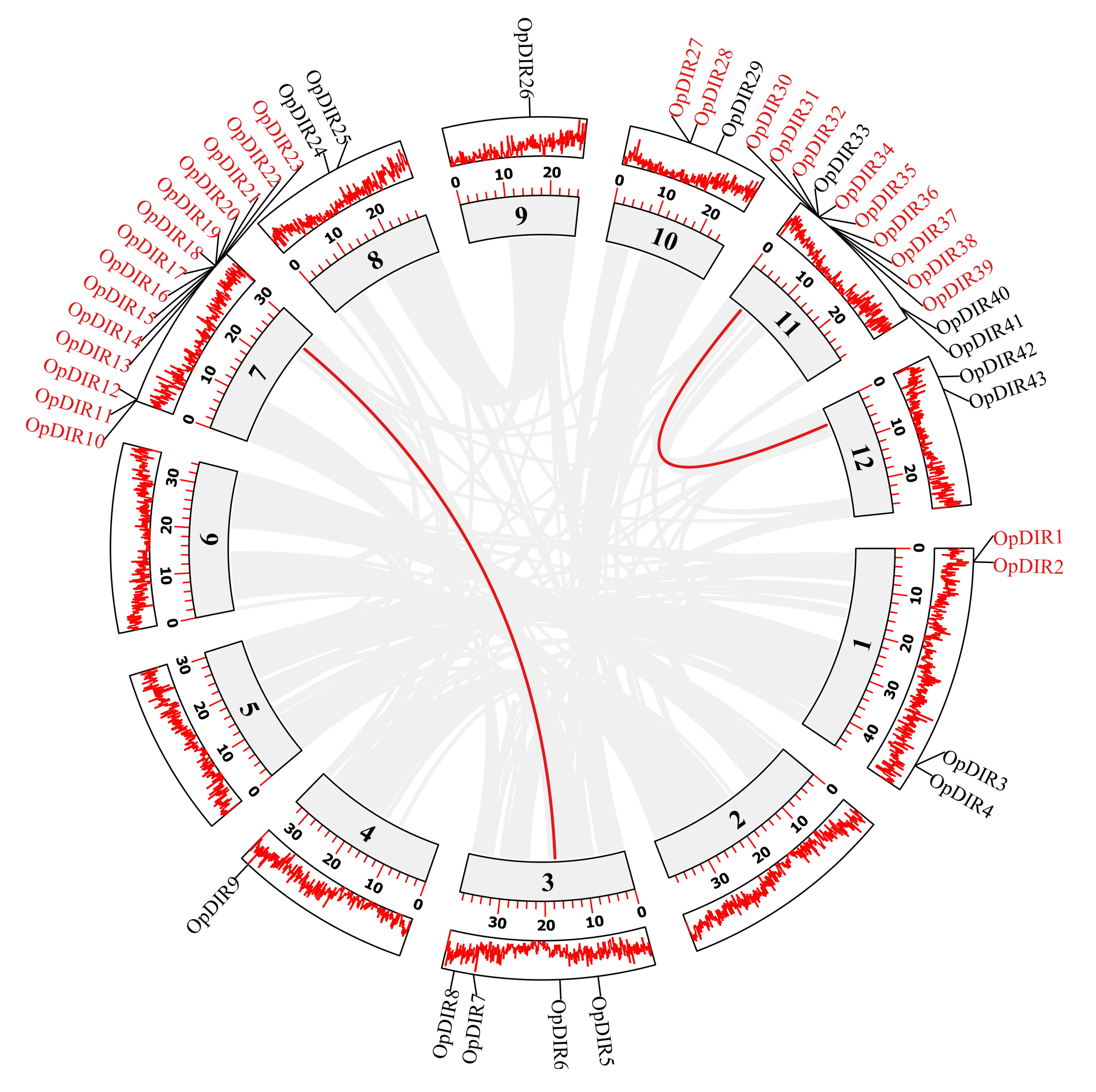

Supplement: Supplementary file 1 [file ijms-24-07189-s001.zip › The location and duplication events of the DIR genes in the genus Oryza/op.tif]

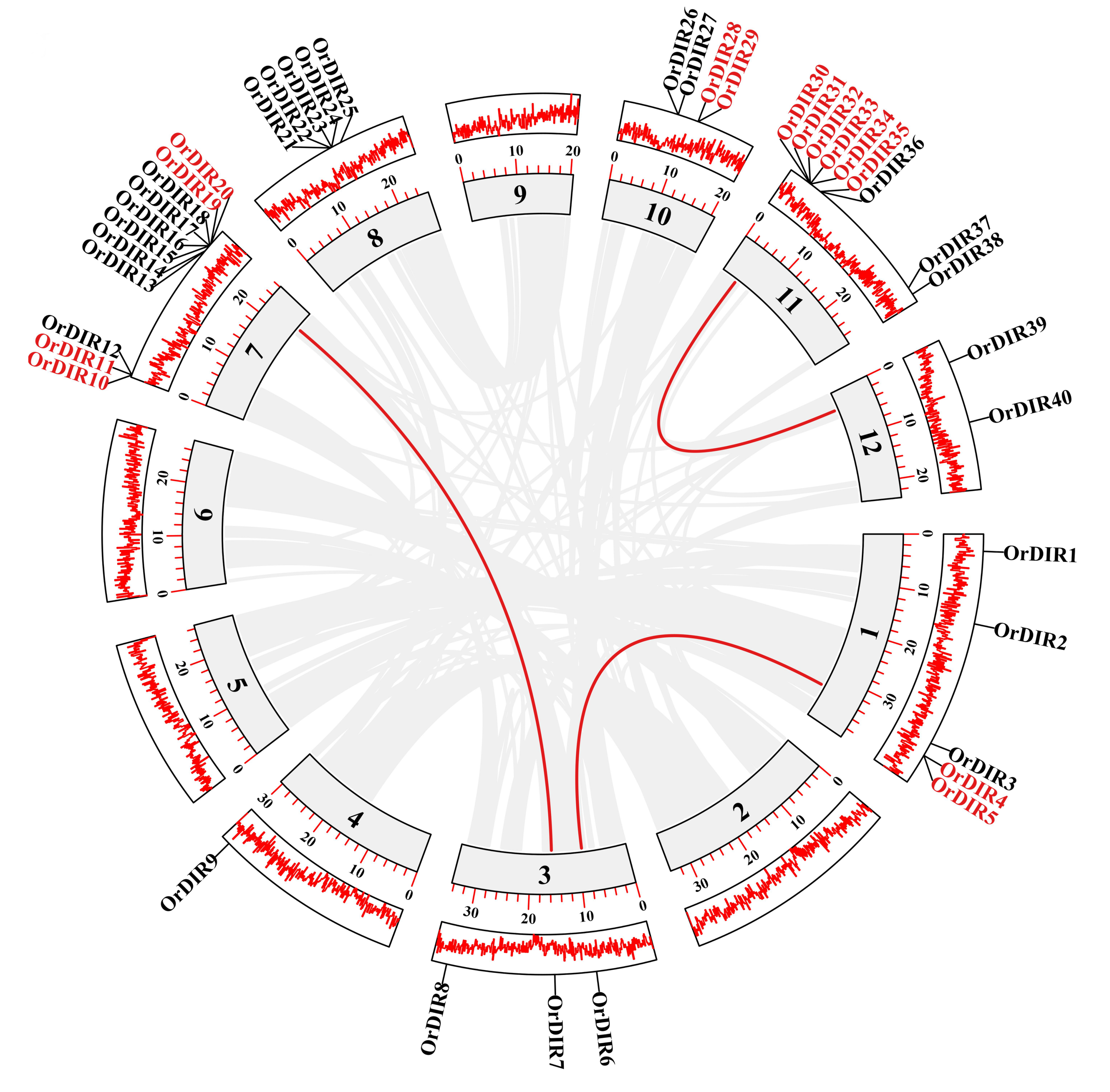

Supplement: Supplementary file 1 [file ijms-24-07189-s001.zip › The location and duplication events of the DIR genes in the genus Oryza/or.tif]

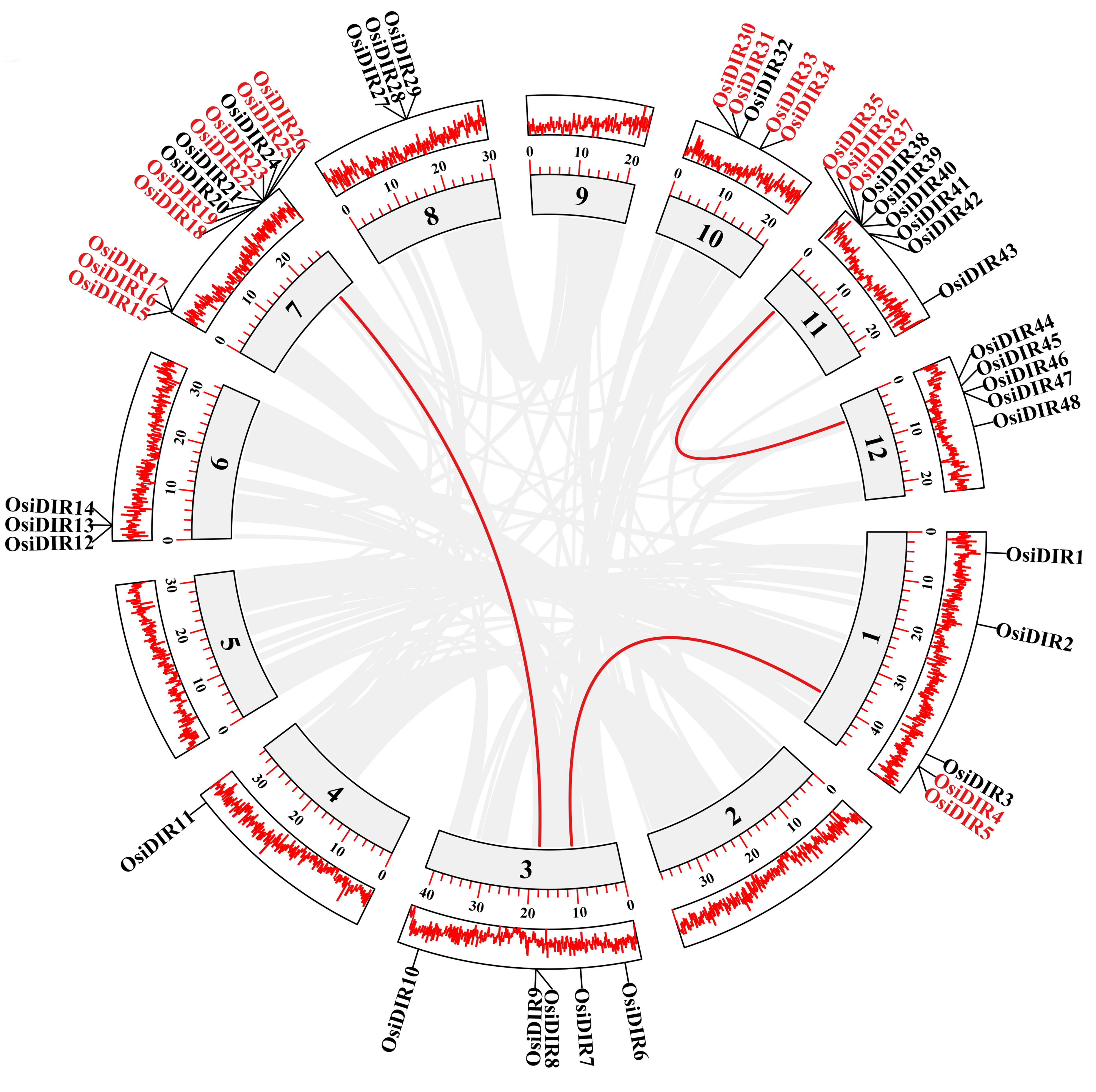

Supplement: Supplementary file 1 [file ijms-24-07189-s001.zip › The location and duplication events of the DIR genes in the genus Oryza/Osi.tif]

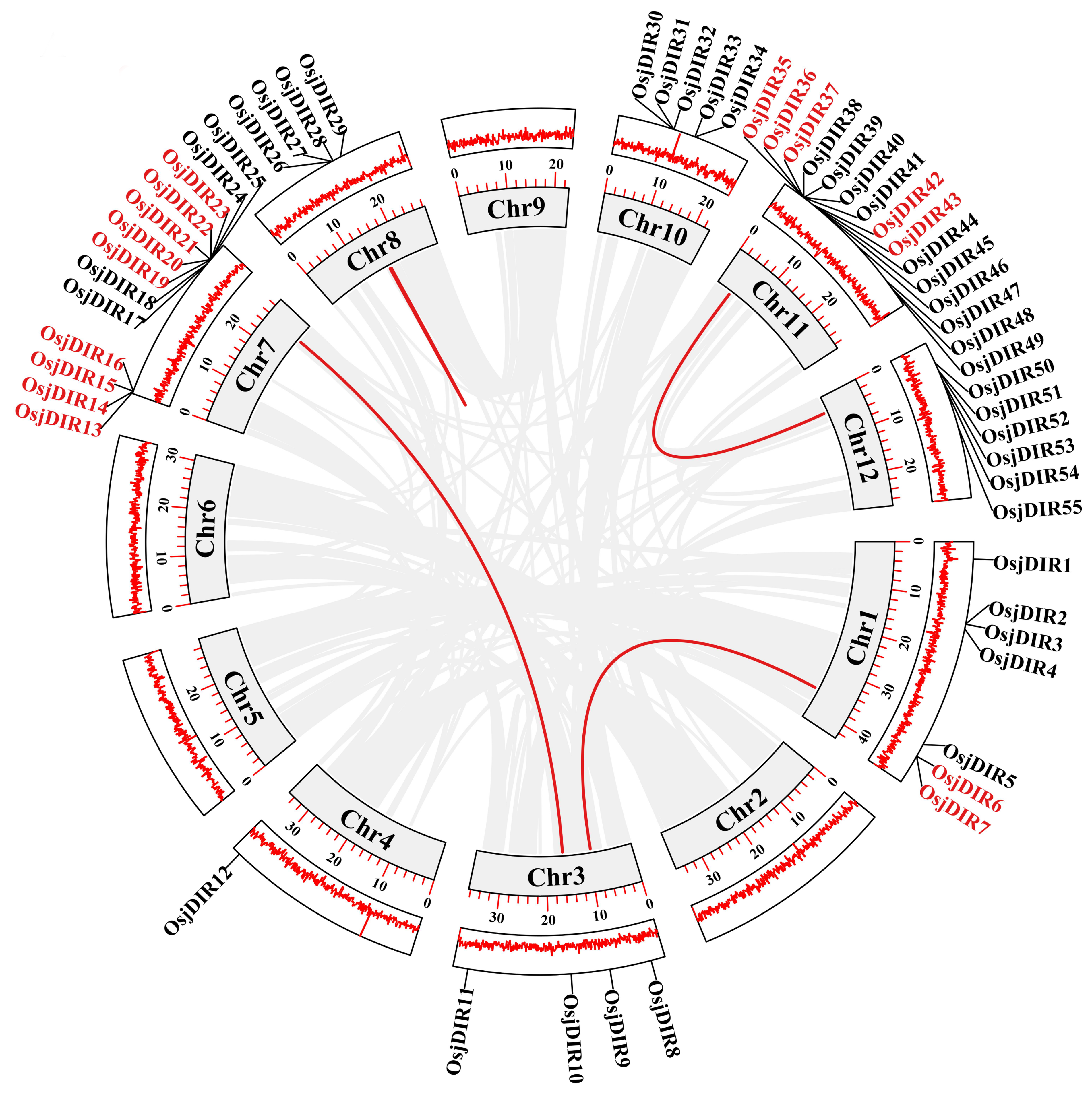

Supplement: Supplementary file 1 [file ijms-24-07189-s001.zip › The location and duplication events of the DIR genes in the genus Oryza/Osj.tif]
